# New Coleoptera records from New Brunswick, Canada: Dermestidae, Endecatomidae, Bostrichidae, and Ptinidae

**DOI:** 10.3897/zookeys.179.2627

**Published:** 2012-04-04

**Authors:** Reginald P. Webster, Jon D. Sweeney, Ian DeMerchant, Martin Turgeon

**Affiliations:** 1 Natural Resources Canada, Canadian Forest Service - Atlantic Forestry Centre, 1350 Regent St., P.O. Box 4000, Fredericton, NB, Canada E3B 5P7; 2 24 Rue Angers, Saint-Basile, New Brunswick, Canada E7C 1V1

**Keywords:** Dermestidae, Endecatomidae, Bostrichidae, Ptinidae, Canada, New Brunswick, new records

## Abstract

We report ten new species records for the Coleoptera fauna of New Brunswick, Canada from the families Dermestidae, Endecatomidae, Bostrichidae, and Ptinidae. *Anthrenus fuscus* Olivier and *Anthrenus museorum* (Linnaeus) of the family Dermestidae are newly recorded for New Brunswick. *Endecatomus rugosus* (Randall) and the family Endecatomidae are recorded for the first time for New Brunswick and the Maritime provinces. Two Bostrichidae, the adventive *Dinoderus minutus* (Fabricius) and the native *Stephanopachys substriatus* (Paykull), are newly recorded for the province. Five species of Ptinidae, the adventive *Anobium punctatum* (DeGeer) and *Microbregma emarginatum emarginatum* (Duftschmid), and the native *Hadrobregmus notatus* (Say), *Ptilinus lobatus* Casey, and *Ptilinus ruficornis* Say are added to the faunal list of New Brunswick. Collection data, habitat data, and distribution maps are presented for all these species.

## Introduction

This paper treats new species records from New Brunswick, Canada of the Coleoptera families Dermestidae, Endecatomidae, Bostrichidae, and Ptinidae. The fauna of these families from New Brunswick and the Maritime provinces (New Brunswick, Nova Scotia, Prince Edward Island) was recently treated by Majka (2007). Intensive sampling in New Brunswick by the first author and others has yielded additional new provincial records in the above families. The purpose of this paper is to report on these new records. A brief synopsis of each family is included in the results below.

## Methods and conventions

The following records are based, in part, on specimens collected as part of a general survey by the first author to document the Coleoptera fauna of New Brunswick and from a study to develop improved lures for survey of potentially invasive species of Cerambycidae. Additional records were obtained from specimens contained in the collection belonging to Natural Resources Canada, Canadian Forest Service - Atlantic Forestry Centre, Fredericton, New Brunswick.

### Collection methods

Various methods were employed to collect the specimens reported in this study. Details are outlined in Webster et al. (2009, Appendix). Many specimens were also collected from Lindgren 12-funnel trap samples during a study to develop a general attractant for the detection of invasive species of Cerambycidae. These traps visually mimic tree trunks and are often effective for sampling species of Coleoptera that live in microhabitats associated with standing trees (Lindgren 1983). See Webster et al. (in press) for details of the methods used to deploy funnel traps and for sample collection. A description of the habitat was recorded for all specimens collected during this survey. Locality and habitat data are presented exactly as on labels for each record. This information, as well as additional collecting notes, is summarized and discussed in the collection and habitat section for each species.

### Distribution

Distribution maps, created using ArcMap and ArcGIS, are presented for each species in New Brunswick. Every species is cited with current distribution in Canada and Alaska, using abbreviations for the state, provinces, and territories. New records for New Brunswick are indicated in bold under Distribution in Canada and Alaska. The following abbreviations are used in the text:

**Table:** 

**AK**	Alaska	**MB**	Manitoba
**YT**	Yukon Territory	**ON**	Ontario
**NT**	Northwest Territories	**QC**	Quebec
**NU**	Nunavut	**NB**	New Brunswick
**BC**	British Columbia	**PE**	Prince Edward Island
**AB**	Alberta	**NS**	Nova Scotia
**SK**	Saskatchewan	**NF & LB**	Newfoundland and Labrador*

*Newfoundland and Labrador are each treated separately under the current Distribution in Canada and Alaska.

Acronyms of collections examined or where specimens reside referred to in this study are as follows:

**AFC** Atlantic Forestry Centre, Natural Resources Canada, Canadian Forest Service, Fredericton, New Brunswick, Canada

**CNC** Canadian National Collection of Insects, Arachnids and Nematodes, Agriculture and Agri-Food Canada, Ottawa, Ontario, Canada

**MTC** Martin Turgeon Collection, Sainte-Basile, New Brunswick, Canada

**NBM** New Brunswick Museum, Saint John, New Brunswick, Canada

**RWC** Reginald P. Webster Collection, Charters Settlement, New Brunswick, Canada

**UMNB **Université de Moncton Collection, Moncton, New Brunswick, Canada

## Results

### Species accounts

All records below are species newly recorded for New Brunswick, Canada. Species followed by ** are newly recorded from the Maritime provinces of Canada.

The classification of the Dermestidae, Endecatomidae, Bostrichidae, and Ptinidae follows Bouchard et al. (2011).

### Family Dermestidae Latreille, 1804

The Dermestidae (skin beetles) are generally scavengers, feeding on dried animal materials such as dried carcasses, old feathers, and plant materials with high protein content (Kingsolver 2002). Some species occur in bee and wasp nests and feed on pollen stores or dried insect remains. A number of species, such as the khapra beetle (*Trogoderma granarium* Everts), are stored-product pests and are serious pests in granaries (Hinton 1945; Kingsolver 1963; Bousquet 1990). Adults of many dermestid species occur on flowers and feed on pollen and nectar (Kingsolver 2002). Majka (2007) reviewed the Dermestidae of the Maritime provinces and reported seven species for New Brunswick. *Attagenus pellio* (Linnaeus), *Attagenus unicolor japonicus* Reitter, and *Dermestes pulcher* LeConte were reported as new. Here, we report two additional species for the province. See Majka (2007) for a list of the other species known from New Brunswick and the other Maritime provinces.

### Subfamily Dermestinae Latreille, 1804

#### Anthrenus fuscus

Olivier, 1789

http://species-id.net/wiki/Anthrenus_fuscus

Map 1

##### Material examined.

**New Brunswick, Madawaska Co.**, St.-Basile, 7.V.1999, M. Turgeon (1, MTC). **Westmorland Co.**, Smith Brook, 21.VII.1995, M. Turgeon (1, MTC).

**Figure:**
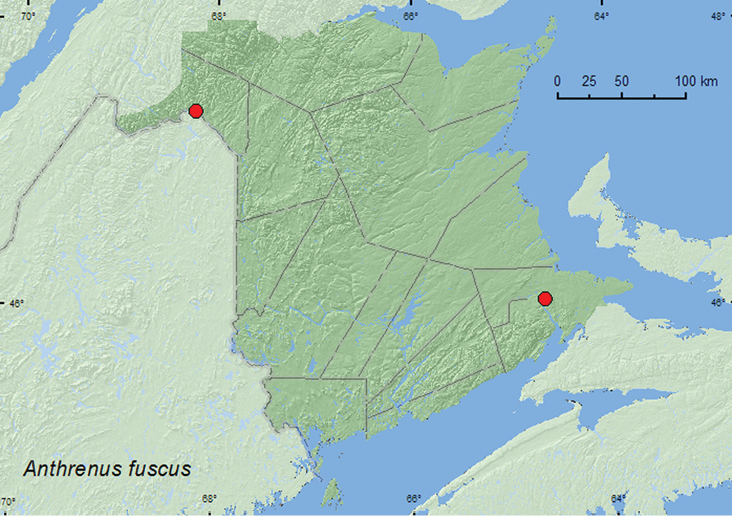
**Map 1.** Collection localities in New Brunswick, Canada of *Anthrenus fuscus*

##### Collection and habitat data.

This adventive Palaearctic species is a minor pest in flour mills, warehouses, and homes (Bousquet 1990) and has been reported from bird and wasp nests, and under bark in natural conditions (Woodroffe and Southgate 1954). Specimens from New Brunswick were collected during May and July, otherwise no other collection data was provided.

##### Distribution in Canada and Alaska.

ON, QC, **NB**, NS, PE (Bousquet 1991a; Majka 2007).

#### Anthrenus museorum

(Linnaeus, 1761)

http://species-id.net/wiki/Anthrenus_museorum

Map 2

##### Material examined.

**New Brunswick, Carleton Co.**, Jackson Falls, Bell Forest, 46.2210°N, 67.7210°W, 25.VI.2007, R. P. Webster, mature hardwood forest, sweeping foliage (1, RWC). **Madawaska Co.**, St.-Basile, 7.V.1999, M. Turgeon, in insect collection (1, MTC); same locality and collector, 29.V.2010 (1, MTC). **York Co.**, Charters Settlement, 45.8395°N, 66.7391°W, 19.VI.2004, 14.VI.2008, R. P. Webster, mixed forest, on flowers of mountain ash and an ornamental *Spiraea* (7, RWC).

**Figure:**
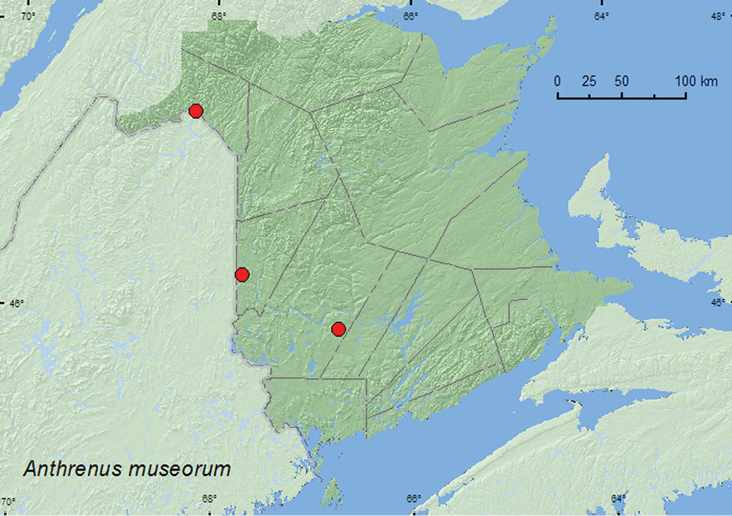
**Map 2.** Collection localities in New Brunswick, Canada of *Anthrenus museorum*.

##### Collection and habitat data.

*Anthrenus museorum* adults were collected from flowers of mountain ash (*Sorbus* sp.) and an ornamental *Spiraea*, and by sweeping foliage in a mature hardwood forest. One adult was found in an insect collection. Adults were captured during May and June. The larvae of this household pest feed on wool, fur, skins, museum specimens, and other animal products. Adults feed on nectar and pollen (Bousquet 1990).

##### Distribution in Canada and Alaska.

ON, QC, **NB,** PE, NS, NF (Bousquet 1991a; Majka 2007).

### Family Endecatomidae LeConte, 1861

The Endecatomidae is a small monogeneric family with four Holarctic species. Lawrence and Newton (1995) treated it as a separate family, but Ivie (2002) treated it as a subfamily of the Bostrichidae, and this reference should be consulted for arguments for and against retaining this subfamily in the Bostrichidae. Here, we follow the classification in Bouchard et al. (2011). Members of this genus feed on fungi (Crowson 1961). *Endecatomus rugosus* (Randall) is the only species of this family recorded from Canada. Here, we record this species and family for the first time from New Brunswick and the Maritime provinces.

#### Endecatomus rugosus

(Randall, 1838)**

http://species-id.net/wiki/Endecatomus_rugosus

Map 3

##### Material examined.

**New Brunswick, Carleton Co.**, Hartland, Becaguimec Island (in Saint John River), 46.3106°N, 67.5372°W, 13.IX.2006, R. P. Webster, old mixed forest, in large dried polypore fungus (1, RWC).

**Figure:**
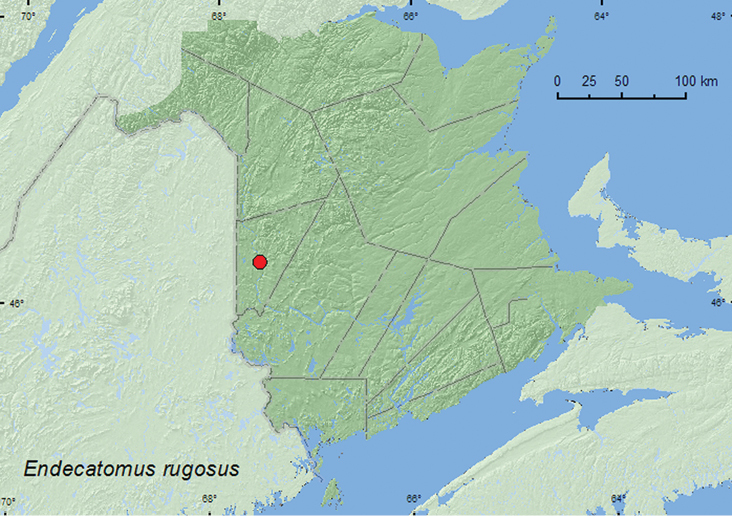
**Map 3.** Collection localities in New Brunswick, Canada of *Endecatomus rugosus*.

##### Collection and habitat data.

One individual of this species was collected from a large old and dried polypore fungus on a partially dead basswood (*Tilia americana* L.) in an old mixed forest.

##### Distribution in Canada and Alaska.

MB, ON, QC, **NB** (McNamara 1991b).

### Family Bostrichidae Latreille, 1802

Larvae of most species of Bostrichidae (the bostrichid beetles) are wood borers and receive their nutrition from the starch content of wood they consume (Gerberg 1957; Ivie 2002). A few species are stored product pests. Those that live in wood typically infest dead and dry wood of angiosperms and dried roots of herbaceous plants and a number of species are subject to distribution around the world by commerce (Ivie 2002). McNamara (1991a, b) reported 23 species of Bostrichidae from Canada. Only one species of Bostrichidae (*Stephanopachys rugosus* (Olivier)) was recorded from New Brunswick by McNamara (1991a). Majka (2007) added the adventive and cosmopolitan *Lyctus brunneus* (Stephens) and *Lyctus linearis* (Goeze) to the faunal list of the province. *Heterobostrychus hamatipennis* (Lesne) was reported from Riverview, Albert Co., New Brunswick, but was considered an intercepted, adventive species that is not established in the region (Majka 2007). Here, we add two additional species to the faunal list of the province. See Majka (2007) for a list of the other species known from New Brunswick and the other Maritime provinces.

### Subfamily Dinoderinae Thomson, 1863

#### Dinoderus minutus

(Fabricius, 1775)

http://species-id.net/wiki/Dinoderus_minutus

Map 4

##### Material examined.

**New Brunswick, Albert Co.**, Riverview Heights, (no day).VIII.1971, (no collector given) ex. carved wood statue and basket (2, AFC).

**Figure:**
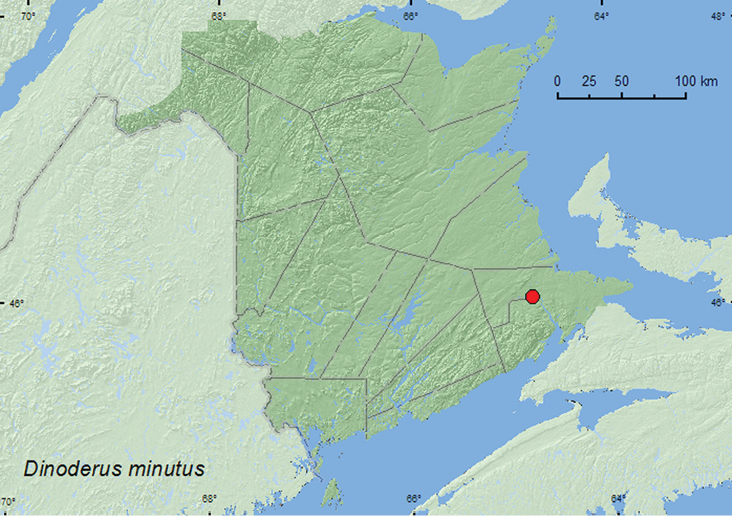
**Map 4.** Collection localities in New Brunswick, Canada of *Dinoderus minutus*.

##### Collection and habitat data.

This adventive species is often found in warehouses and places where bamboo products are stored (Bousquet 1990). Adult specimens from New Brunswick emerged from a carved wood statue and basket. This species develops in bamboo in the tropics but can be found in dried food products in North America (Spillman 1982). It is not clear if this species is established in New Brunswick.

##### Distribution in Canada and Alaska.

BC, SK, MB, ON, **NB**,PE (Bousquet 1990; Majka 2007).

#### Stephanopachys substriatus

(Paykull, 1800)

http://species-id.net/wiki/Stephanopachys_substriatus

Map 5

##### Material examined.

**New Brunswick, Sunbury Co.**, Acadia Research Forest, 45.9866°N, 66.3841°W, 9–16.VI.2009, R. Webster & M.-A. Giguère, mature (110-year-old) red spruce forest with scattered red maple and balsam fir, Lindgren funnel trap (1, RWC). **York Co.**, 15 km W of Tracy off Rt. 645, 45.6848°N, 66.8821°W, 15–21.VI.2010, R. Webster & M.-A. Giguère, old red oak forest, Lindgren funnel trap (1, AFC).

**Figure:**
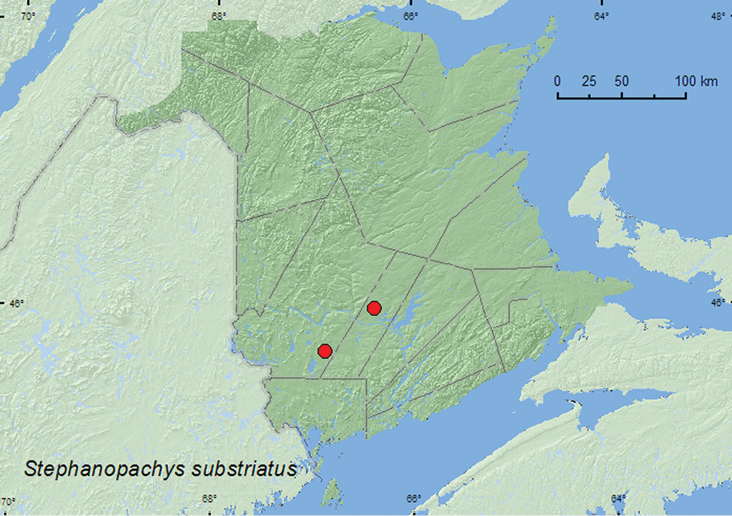
**Map 5.** Collection localities in New Brunswick, Canada of *Stephanopachys substriatus*.

##### Collection and habitat data.

The two individuals from New Brunswick were captured during June in Lindgren funnel traps in a red spruce (*Picea rubens* Sarg.) forest and an old red oak (*Quercus rubra* L.) forest. Members of this genus are associated with conifers (Ivie 2002).

##### Distribution in Canada and Alaska.

AK, YK, NT, BC, AB, MB, ON, QC, **NB**, NS (McNamara 1991a).

### Family Ptinidae Latreille, 1802

The Ptinidae (death watch and spider beetles) (formerly Anobiidae) are borers in bark, dry wood, twigs, galls, pine cones, and fungi (many Anobiinae) or feed on accumulated dried animal and plant material, and are found in bird, mammal, and solitary bee nests (mostly Ptininae) (Hinton 1941; Philips 2002). A number of species are important pests and have been widely distributed by commerce around the world. The furniture beetle, *Anobium puncatum* (DeGeer), causes damage to furniture, woodwork of houses, and books (Philips 2002). The drugstore beetle, *Stegobium paniceum* (Linnaeus), and the cigarette beetle, *Lasioderma serricorne* (Fabricius), are important stored-products pests (tobacco, spices, cayenne pepper) (Philips 2002). McNamara (1991a) and Bousquet (1991b) reported 95 species of Ptinidae from Canada and 17 species from New Brunswick. In a recent review of the Ptinidae (as Anobiidae) of the Maritime provinces, Majka (2007) newly recorded the adventive *Ernobius mollis* (Linnaeus), *Lasioderma serricorne* (Fabricius), and *Ptinus clavipes* (Panzer) from New Brunswick, bringing the total number of species known from the province to 20. Here, we newly report another five species from the province. See Majka (2007) for a list of the other species known from New Brunswick and the other Maritime provinces.

### Subfamily Anobiinae Fleming, 1821

#### Anobium punctatum

(DeGeer, 1774)

http://species-id.net/wiki/Anobium_punctatum

Map 6

##### Material examined.

**New Brunswick, Kent Co.**, Richibucto, 2.VII.1989, P. Maltais (1, UMNB). **Kings Co.**, Sussex, 13.IX.1957, C.C. Smith, from barn timbers (3, AFC). **York Co.**, Fredericton, 24.I.1934, ex. seasoned wood (3, AFC).

**Figure:**
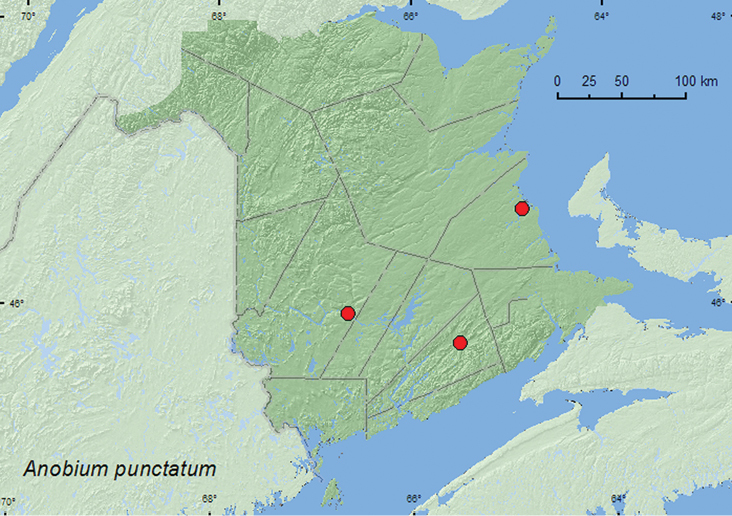
**Map 6.** Collection localities in New Brunswick, Canada of *Anobium punctatum*.

##### Collection and habitat data.

Known as the furniture beetle, this adventive species feeds on a variety of soft- and hardwood species (Philips 2002). Adults from New Brunswick emerged from barn timbers and seasoned wood.

##### Distribution in Canada and Alaska.

BC, AB, MB, QC, **NB**, NS, NF (McNamara 1991a).

#### Microbregma emarginatum emarginatum

(Duftschmid, 1825) 

http://species-id.net/wiki/Microbregma_emarginatum_emarginatum

Map 7

##### Material examined.

**New Brunswick, Carleton Co.**, Jackson Falls, Bell Forest, 46.2200°N, 67.7231°W, 12–19.VI.2008, 27.VI–5.VII.2008, R. P. Webster, mature hardwood forest, Lindgren funnel traps (2, RWC). **Restigouche, Co.**, Dionne Brook P.N.A. (Protected Natural Area), 47.9064°N, 68.3441°W, 31.V–15.VI.2011, 15–27.VI.2011, M. Roy & V. Webster, old-growth white spruce and balsam fir forest, Lindgren funnel traps (7, AFC, NBM, RWC). **York Co.**, 14 km WSW of Tracy, S of Rt. 645, 45.6741°N, 66.8661°W, 10–26.V.2010, 2–16.VI.2010, R. Webster & C. MacKay, old mixed forest with red and white spruce, red and white pine, balsam fir, eastern white cedar, red maple, and *Populus* sp., Lindgren funnel trap (2, RWC); 15 km W of Tracy off Rt. 645, 45.6848°N, 66.8821°W, 8–20.VI.2011, M. Roy & V. Webster, old red pine forest, Lindgren funnel traps (3, AFC, RWC).

**Figure:**
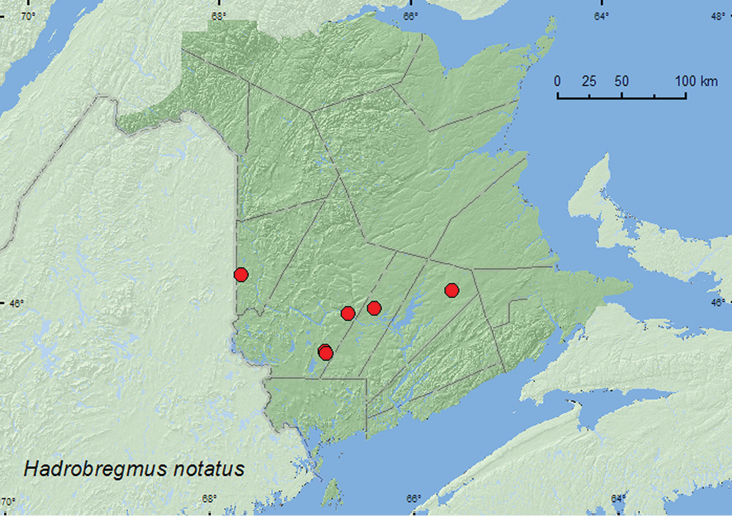
**Map 8.** Collection localities in New Brunswick, Canada of *Hadrobregmus notatus*.

##### Collection and habitat data.

In New Brunswick, adults of this species were captured in Lindgren funnel traps in a hardwood forest with sugar maple (*Acer saccharum* Marsh.), American beech (*Fagus grandifolia* Ehrh.), white ash (*Fraxinus americana* L.), and small areas of eastern hemlock (*Tsuga canadensis* (L.) Carr.) and eastern white cedar (*Thuja occidentalis* L.), an old mixed forest with red spruce, white spruce (*Picea glauca* (Moench) Voss), red pine (*Pinus resinosa* Ait.), white pine (*Pinus strobus* L.), balsam fir (*Abies balsamea* (L.) Mill., eastern white cedar, red maple (*Acer rubrum* L.), and *Populus* sp., an old red pine forest, and an old-growth white spruce and balsam fir forest. White (1982) reported this adventive Palaearctic species from under bark of pine, hemlock, and hickory (*Carya* sp.). Adults were captured during May and June in New Brunswick.

##### Distribution in Canada and Alaska.

BC, AB, SK, MB, ON, QC, **NB**, PE, NS (McNamara 1991a; Majka 2007).

#### Hadrobregmus notatus

(Say, 1825)

http://species-id.net/wiki/Hadrobregmus_notatus

Map 8

##### Material examined.

**New Brunswick, Carleton Co.**, Jackson Falls, Bell Forest, 46.2200°N, 67.7231°W, 12–19.VI.2008, R. P. Webster, mature hardwood forest, Lindgren funnel trap (1, RWC). **Queens Co.**, Cranberry Lake P.N.A., 46.1125°N, 65.6075°W, 18–25.VI.2009, 15–21.VII.2009, 28.VII–6.VIII.2009, R. Webster & M.-A. Giguère, mature red oak forest, Lindgren funnel traps (5, AFC, RWC). **Sunbury Co.**, Acadia Research Forest, 45.9866°N, 66.3841°W, 19–25.V.2009, 9–16.VI.2009, 16–24.VI.2009, 24–30.VI.2009, 30.VI-8.VII.2009, R. Webster & M.-A. Giguère, mature (110-year-old) red spruce forest with scattered red maple and balsam fir, Lindgren funnel traps (6, AFC, RWC). **York Co.**, Fredericton, 9.X.1958, C. C. Smith, ex. *Picea glauca* (2, AFC); 15 km W of Tracy off Rt. 645, 45.6848°N, 66.8821°W, 15–21.VI.2010, 21–28.VI.2009, 28.VI–7.VII.2009, 14–20.VII.2009, 20–29.VII.2009, 11–18.VIII.2009, R. Webster & M.-A. Giguère, old red pine forest, Lindgren funnel traps (9, AFC, RWC); 14 km WSW of Tracy, S of Rt. 645, 45.6741°N, 66.8661°W, 16–30.VI.2010, R. Webster & C. MacKay, old mixed forest with red and white spruce, red and white pine, balsam fir, eastern white cedar, red maple, and *Populus* sp., Lindgren funnel traps (2, AFC).

**Figure:**
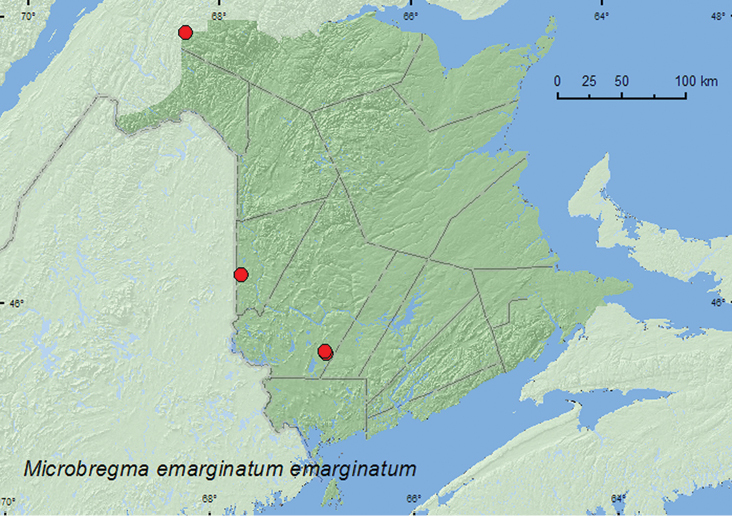
**Map 7.** Collection localities in New Brunswick, Canada of *Microbregma emarginatum emarginatum*.

##### Collection and habitat data.

In New Brunswick, this species was captured in Lindgren funnel traps in a mature hardwood forest with sugar maple, American beech, and white ash, an old red oak forest, an old mixed forest, a mature red spruce forest with scattered red maple and balsam fir, and an old red pine forest. Two individuals were reared from white spruce. White (1982) reported this species from dead and rotten oak, ash, pine, and pine boards. Adults were captured during June, July, and August in New Brunswick.

##### Distribution in Canada and Alaska. 

ON, QC, **NB**, PE, NS (McNamara 1991a; Majka 2007).

### Subfamily Ptilininae Shuckard, 1839

#### Ptilinus lobatus

Casey, 1898

http://species-id.net/wiki/Ptilinus_lobatus

Map 9

##### Material examined.

**New Brunswick, Carleton Co.**, Meduxnekeag Valley Nature Preserve, 46.1931°N, 67.6825°W, 20.VI.2005, M.-A. Giguère, floodplain forest with butternut, on trunk of *Prunus serotina* (2, RWC); Jackson Falls, Bell Forest, 46.2200°N, 67.7231°W, 21–28.VI.2009, R. Webster & M.-A. Giguère, mature hardwood forest, Lindgren funnel trap (1, AFC). **Queens Co.**, Cranberry Lake P.N.A., 46.1125°N, 65.6075°W, 1–10.VII.2009, R. Webster & M.-A. Giguère, old red oak forest, Lindgren funnel trap (1, RWC).

**Figure:**
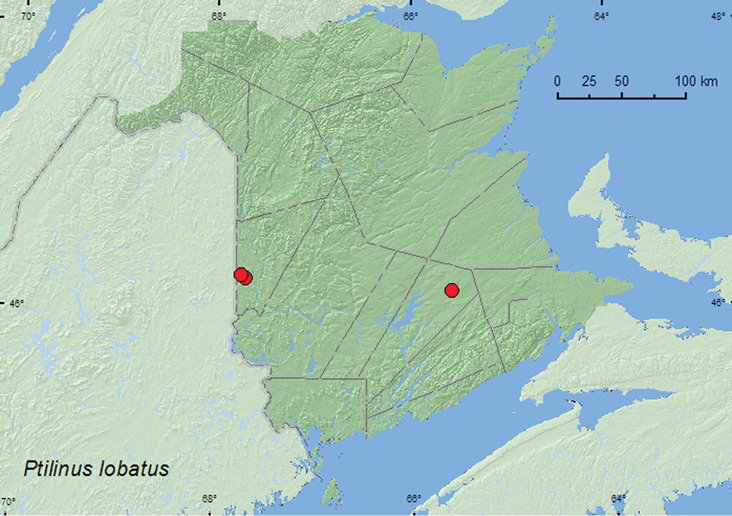
**Map 9.** Collection localities in New Brunswick, Canada of *Ptilinus lobatus*.

##### Collection and habitat data. 

Specimens from New Brunswick were captured from Lindgren funnel traps in a mature hardwood forest with sugar maple, American beech, and white ash, and an old red oak forest. Two specimens were collected from the trunk of a black cherry (*Prunus serotina* Ehrh.) in a floodplain forest with butternut (*Juglans cinerea* L.). The larvae of *Ptilinus* species mine hardwoods (Philips 2002). Majka (2007) reported the species from *Populus* logs in Nova Scotia. Adults were captured during June and July in New Brunswick.

##### Distribution in Canada and Alaska. 

YK, BC, AB, MB, ON, **NB**, NS (McNamara 1991a; Majka 2007).

#### Ptilinus ruficornis

Say, 1823

http://species-id.net/wiki/Ptilinus_ruficornis

Map 10

##### Material examined.

**New Brunswick, Carleton Co.**, North Richmond (now probably Richmond Corner), 20.VI.1942, R. E. Currie, beating balsam fir foliage, F.I. Survey 42-L113 (1, AFC); Jackson Falls, Bell Forest, 46.2200°N, 67.7231°W, 5–12.VII.2008, R. P. Webster, mature hardwood forest, Lindgren funnel trap (1, RWC); same locality and habitat data but 21–28.VI.2009, 28.VI–7.VII.2009, R. Webster & M.-A. Giguère, Lindgren funnel traps (9, AFC, RWC). **Charlotte Co.**, 10 km NW of New River Beach, 45.2110°N, 66.6170°W, 29.VI-16.VII.2010, R. Webster & C. MacKay, old growth eastern white cedar forest, Lindgren funnel trap (1, AFC). **Queens Co.**, Cranberry Lake P.N.A., 46.1125°N, 65.6075°W, 18–25.VI.2009, 10–15.VII.2009, R. Webster & M.-A. Giguère, old red oak forest, Lindgren funnel traps (2, RWC); Grand Lake Meadows P.N.A., 45.8227°N, 66.1209°W, 19.VII–5.VIII.2011, M. Roy & V. Webster, old silver maple forest with green ash and seasonally flooded marsh, Lindgren funnel trap (1, NBM). **Saint John Co.**, Fairville Plateau, 30.VI.1949, from house, (9, AFC). **Sunbury Co.**, Acadia Research Forest, 45.9866°N, 66.3841°W, 24–30.VI.2009, R. Webster & M.-A. Giguère, mature (110-year-old) red spruce forest with scattered red maple and balsam fir, Lindgren funnel trap (1, RWC). **York Co.**, 15 km W of Tracy off Rt. 645, 45.6848°N, 66.8821°W, 15–21.VI.2009, 21–28.VI.2009, 28.VI–7.VII.2009, R. Webster & M.-A. Giguère, old red pine forest, Lindgren funnel traps (3, AFC); same locality and habitat data, 16–30.VI.2010, 30.VI–13.VII.2010, R. Webster, C. MacKay, & K. Burgess, Lindgren funnel traps (3, AFC, RWC).

**Figure:**
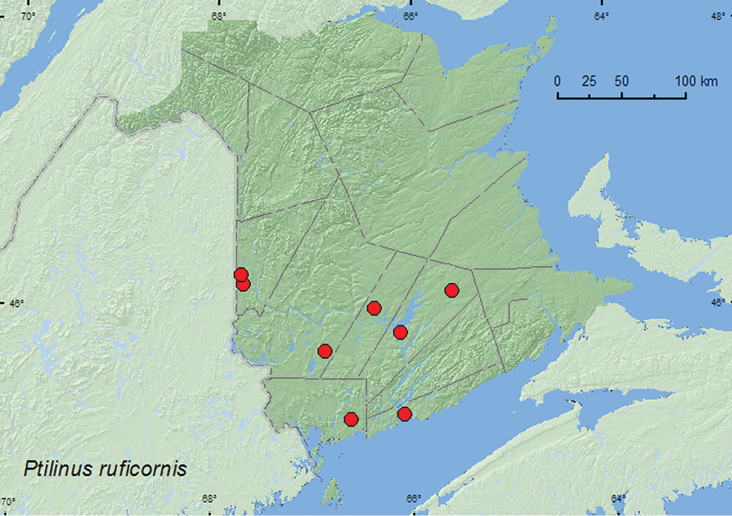
**Map 10.** Collection localities in New Brunswick, Canada of *Ptilinus ruficornis*.

##### Collection and habitat data.

*Ptilinus ruficornis* occurred in various deciduous and coniferous forest types in New Brunswick. This species was captured in a mature hardwood forest with sugar maple, American beech, and white ash, an old red oak forest, an old silver maple (*Acer saccharinum* L.) forest, an old red pine forest, a mature (110-year-old) red spruce forest with scattered red maple and balsam fir, and an old-growth eastern white cedar forest/swamp. Most adults were captured in Lindgren funnel traps; one individual was beaten from balsam fir foliage. The larvae of *Ptilinus* sp. mine hardwoods (Philips 2002). Adults were captured during June, July, and August.

##### Distribution in Canada and Alaska.

AB, ON, QC, **NB**, NS (McNamara 1991a).

## Supplementary Material

XML Treatment for Anthrenus fuscus

XML Treatment for Anthrenus museorum

XML Treatment for Endecatomus rugosus

XML Treatment for Dinoderus minutus

XML Treatment for Stephanopachys substriatus

XML Treatment for Anobium punctatum

XML Treatment for Microbregma emarginatum emarginatum

XML Treatment for Hadrobregmus notatus

XML Treatment for Ptilinus lobatus

XML Treatment for Ptilinus ruficornis
